# How individuals perceive their partner’s relationship behaviors when worrying about finances

**DOI:** 10.1177/02654075241227454

**Published:** 2024-01-17

**Authors:** Johanna Peetz, Odin Fisher-Skau, Samantha Joel

**Affiliations:** 1 6339 Carleton University, Canada; 2 6221 University of Western Ontario, Canada

**Keywords:** Financial stress, financial worry, interpersonal perception, perception accuracy, relationship behaviors, truth and bias model

## Abstract

What role do financial worries play in close relationship functioning? In this research, we examine how financial worry – negative thoughts and feelings about finances – is associated with perceived relationship behaviors. Participants recalled how their partner acted during a recent disagreement (Study 1, *N* = 97 couples) or recalled the frequency of positive and negative behaviors enacted by their partner during the previous week (Study 2, *N* = 99 couples). Feeling more worried about finances was associated with recalling less supportive behavior from one’s partner at the disagreement (Study 1) and with perceiving more negative behaviors from one’s partner in the last week (Study 2). Truth and Bias Model analyses suggest that part of this link may be attributed to biased perceptions, as the link between financial worry and perceiving more negative behaviors persisted even after controlling for participants’ own reported behaviors (i.e., accounting for similarity) and for their partner’s own reported behaviors (i.e., accounting for accurate perceptions). In sum, financial worry is linked to how partners notice and interpret a loved one’s actions within their relationship.

## Introduction

World-wide, people are experiencing economic uncertainty and an affordability crisis as result of the pandemic and recession (e.g., APA, 2023). Financial stress and financial hardship have been linked to poorer relationship quality (e.g., Dew, 2008; Falconier & Jackson, 2020; Jackson et al., 2023; Kelley et al., 2018; Kerkmann et al., 2000; Totenhagen et al., 2019). In the current research, we examine one potential correlate of financial stress and ruminating about one’s finances: the extent to which relationship partners perceive positive and negative relationship behaviors from their loved one. Financial stress can affect cognitive functioning, impact individuals’ memory (e.g., Gennetian & Shafir, 2015), attentional allocation (e.g., Mani et al., 2020), and perception of social cues (e.g., Park et al., 2017), all of which might affect relationship behaviors and perceptions thereof. We examine whether financial worries are associated with perceiving fewer positive behaviors (such as acting supportive during a disagreement, physical affection, support, and help) and more negative behaviors (such as neglecting chores, saying things that hurt the partner’s feelings) from one’s relationship partner.

### Worrying about finances

According to the Gallup Index, 40–52% of U.S. Americans reported being moderately or very worried about not having enough money to pay normal monthly bills (Saad, 2022) and 66% reported that money is a significant source of stress (APA, 2023). In the present set of studies, we examine the subjective worry about finances, regardless of objective financial stressors. We define financial worry as repeated negative thinking about one’s financial situation (de Bruijn & Antonides, 2020), including both negative *emotions* such as anger, being upset or anxious about finances and negative *cognitions* such as repeated and interfering thoughts about one’s financial situation. Although subjective financial stress and worry about finances can be found at any income level, they have been linked to objective financial stressors such as income or income volatility, with people at the lower end of the income distribution being more prone to worry and ruminate about finances (Johar et al., 2015; Magwegwe et al., 2023).

### Relationship behaviors

High-quality relationships are characterized by relatively more positive relationship behaviors that make the partner feel understood, appreciated, and supported (e.g., Gable & Reis, 2010; Reis et al., 2004) and relatively fewer relationship behaviors that threaten, undermine, or hurt the partner (e.g., Goodboy & Myers, 2010). Examples of positive relationship behaviors include self-disclosure (Sprecher & Hendrick, 2004), arranging date nights (Girme et al., 2014), completing household chores (Newkirk et al., 2017), and engaging in self-expanding activities with the partner (Muise et al., 2019). Examples of negative behaviors include blame, rejection (Rogge & Bradbury, 1999), criticism, or holding the partner in poor regard (Murray et al., 2003).

Notably, *perceptions* of such behaviors tend to be more strongly linked to relationship quality than *actual* relationship behaviors as reported by the partner (e.g., Dobson et al., 2021; Joel et al., 2022; Reis et al., 2004). For example, people who feel more satisfied with their relationships tend to expect and, in turn, perceive more positive behaviors and fewer negative behaviors from their partners, over and above their partners’ reports of their own behaviors (Joel et al., 2022). The present studies examine the role of financial stress and worry in the *perception* of relationship behaviors.

#### Financial worry and relationship behaviors

A well-established consequence of financial stress is lower overall well-being, such as worse mental health (see Guan et al., 2022; Selenko & Batinic, 2011; for reviews). In a sample of Black single mothers, when providing reasons for their depressed mood, the most frequent responses referenced financial stressors (Atkins et al., 2020). Financial stress was also a major determining factor of personal well-being during the COVID-19 pandemic (Hertz-Palmor et al., 2021; Swigonski et al., 2021). At the opposite end of the spectrum, earning *more* money has been linked to less daily sadness (Kushlev et al., 2015), and positive and negative affect are strongly associated with increased and decreased financial satisfaction, respectively (Tharp et al., 2020). Thus, there is extensive evidence that financial stress and financial insecurity go hand in hand with negative emotions.

Diminished mood might lead people to perform fewer supportive and more negative behaviors towards their partner. Indeed, in a sample of unemployed job seekers and their partners, financial strain was linked to depressive symptoms in both partners (Vinokur et al., 1996). Furthermore, although there was no significant direct link between financial strain and relationship behaviors, depressive symptoms were linked to fewer positive relationship behaviors (withdrawal of social support) and more negative relationship behaviors (undermining behaviors) by the job seeker’s partner (Vinokur et al., 1996). Beyond the domain of finances, experiencing stress and depressed mood has been linked to romantic partners reporting having perfomed fewer positive relationship behaviors (Neff et al., 2021) and more negative relationship behaviors (Repetti, 1989) themselves. Newlyweds who experienced more stress in the previous seven months across 13 life domains reported providing less support to their spouse across multiple diary assessments (Neff et al., 2021). Employed partners who felt busy and overloaded at work reported worse mood and, in turn, reported more negative relationship behaviors (anger, disregard, and distancing) (Repetti, 1989).

#### Financial worry and perception

Financial stress has also been associated with lower cognitive function (Gennetian & Shafir, 2015; Shafir, 2017; Sheehy-Skeffington, 2020). For example, poverty indicators were linked with worse Stroop task performance (Mani et al., 2013) and income uncertainty was linked to lower working memory and reduced ability to pay attention (Lichand & Mani, 2020). Ruminating about one’s finances likely distracts and distorts perception, just like negative emotions and worries of any kind have been associated with impaired cognitive functioning (see Nolen-Hoeksema et al., 2008, for an overview).

On one hand, the cognitive distraction of ruminating about finances might lead people to notice less of what their loved ones do – thus perceiving both fewer positive and fewer negative behaviors. On the other hand, feeling stressed, upset, angry, anxious, or insecure about one’s finances might colour perceptions such that a person who is worried about their finances might perceive a close other’s behaviors in a more negative light, perceiving *fewer* positive behaviors but perceiving *more* negative behaviors. Both possibilities include perceiving fewer positive behaviors when feeling stressed about finances, and indeed, data from the National Survey of Midlife Development in the United States showed that people who were more dissatisfied with their financial situation perceived less social support from a range of close others, including friends, family, and significant others (Park et al., 2017).

Negative feelings such as general stress have been shown to affect individuals’ perception of their significant other’s behaviors (Crenshaw et al., 2019; Neff & Buck, 2023; Overall & Hammond, 2013). For instance, participants who reported more depressive symptoms perceived more negative behaviors from their partner over the course of three weeks than their partner reported performing (Overall & Hammond, 2013). Newlyweds who experienced more stressful life events (including stressors related to marriage, work, school, health, personal events, living condition, legal action, and finances) in the past six months likewise perceived more negative relationship behaviors from their partner across a 10-day period than their partners reported performing (Neff & Buck, 2023). In the present studies we examine whether similar patterns occur for how *financial* worry – negative emotions and thoughts specific to one’s financial situation – is associated with perception of a romantic partner’s relationship behaviors, while examining positive and negative relationship behaviors separately.

### Overview of studies

Across two dyadic studies we examined the research question, “How does financial worry affect perception of the partner’s relationship behaviors?”. In the initial study, we examined the association between participants’ own financial worry and participants’ perception of their partner acting supportive during a recent disagreement. In a second study, we examined the association between participants’ financial worry and the frequency of positive and negative relationship behaviors they perceived their partner performing in the last week. In this study, participants and their respective partners also reported their own relationship behaviors, allowing us to examine multiple perspectives of how many behaviors were performed. We hypothesized that:

Hypothesis 1 Financial worry would be associated with perceiving fewer supportive behaviors during a disagreement and fewer positive behaviors overall.

Hypothesis 2a Financial worry might be associated with perceiving *fewer* negative behaviors overall as the cognitive distraction accompanying rumination about finances (Shafir, 2017; Sheehy-Skeffington, 2020) might prevent noticing, encoding, or recalling any partner behaviors.

Hypothesis 2b Financial worry might be associated with perceiving *more* negative behaviors overall as the negative mood accompanying financial stress (Selenko & Batinic, 2011) might negatively bias which behaviors are noticed or how behaviors are interpreted.

These hypotheses draw from dominant theories about stress, such as the Family Stress model (Conger et al., 1994, 2010)—which outlines the impact of economic stressors on families—and the Vulnerability-Stress-Adaptation model (Karney & Bradbury, 1995), which outlines the impact of external stressors on marital quality via (mal)adaptive processes. Consistent with these theoretical perspectives, we expected subjective economic stress to shape perceptions of relationship behaviors. The present studies also extend prior research on links between relationship behaviors and stress (Crenshaw et al., 2019; Neff & Buck, 2023; Overall & Hammond, 2013) to the domain of financial stress and financial worry specifically. Financial worry might be particularly influential for relationship processes because finances of individuals in a relationship are often interdependent, whereby one partner’s spending decisions affect both partners’ financial situation. A person’s financial worry may affect their behaviors towards the other person and their perception of the other person’s behavior. The present studies aim to tease apart how each partner’s financial worry might be linked to a specific relationship cognition: perception of positive and negative relationship behaviors.

## Study 1

In this first study, we examined perception of supportive behavior during a specific interaction with the partner. We asked participants to rate their perception of their partner’s supportive behavior during a recent disagreement. We also assessed overall appraisal of the relationship. Unabbreviated materials, data, and syntax are available on OSF: https://osf.io/hq8ba/. This study was not preregistered. We report all manipulations, measures, and exclusions in this study.

### Method

#### Participants

A total of 217 participants in relationships who also had a partner with access to the data collection platform were recruited from Prolific Academic and compensated with $10 (prorated for $20/hour). Participants passed Prolific data quality checks and a reCAPTCHA check before starting the study as well as an attention check during the study. After an initial set of participants completed the survey, nominating a recent disagreement along with keywords describing the disagreement and date(s) of the disagreement, we contacted participants’ partners to invite them to complete the survey as well. For our analyses, we designated the first partner, who nominated the disagreement, as “actor”, and their corresponding partner as “partner”. Partners were asked to recall and answer questions about the same disagreement based on keywords and date(s) provided by their partners. We included only datasets where both partners completed the survey, resulting in a final sample of 200 participants (100 couples). Three couples were excluded from this dataset because the disagreements they reported on did not match (as judged by a research assistant who read the full descriptions of the interaction by both participants), resulting in a final sample of 194 participants (97 couples). Less than .01% of data was coded missing. Simulated power analyses (Lane & Hennes, 2018) suggest that this sample has 91.6% power to detect a small effect (*b* = .20), and over 99% power to detect a medium (*b* = .35) or larger effect in the context of a multilevel model with two predictors.

The sample included 99 women and 95 men, ranging in age from 22 to 85 years (*M* = 37.91 years, *SD* = 10.13, *Mdn* = 36). The majority of the sample was White (90.2% White, 3.1% Black, 3.1% of Asian descent, 3.6% mixed heritage). Most participants were from the U.K. (93%), with the remaining participants from the US (3%) and Canada (4%). The majority (92.5%) considered themselves heterosexual or straight, 1% considered themselves gay, 1% lesbian, 4.5% bisexual, 0.5% queer, and 0.5% pansexual. Almost all couples (96.9%) were married or living in a common-law marriage (50.5% of the couples were married, 11.9% were engaged and living together, 34.5% were dating and living together at least a year [i.e., common-law], and 3.1% were dating and not living together). Average relationship length was 11.75 years (*SD* = 7.9). We did not assess student status or disability status. Participants’ annual gross income ranged from “under $10,000” to “$100,000 or more”, with the median income category being “$40,000-$50,000”, around which the income was normally distributed. For comparison, the average annual income for U.S. workers is approximately $60,575 and the median income is $56,420 (DeMarco, 2023), suggesting that our sample earned just a little below the national average.

#### Procedure

Participants completed a brief demographic survey (age, gender, ethnicity, relationship status and length) and reported on their financial situation (income, income volatility). Participants reported financial stress on one item (Frone et al., 1992, “Right now, how stressed do you feel about your financial situation?”), using a Likert scale ranging from 1 = *Not stressed at all* to 10 = *Extremely stressed*. In addition, we assessed a single item on financial satisfaction (Plagnol, 2011; Voydanoff, 2004; “Overall, how satisfied are you with your household’s financial situation?”), using a Likert scale ranging from 1 = *Very dissatisfied* to 7 = *Very satisfied,* and a single item on financial worry (Voydanoff, 2004; “How often do you worry that your total family income will not be enough to meet your family’s expenses and bills? Would you say.... ”), using a Likert scale ranging from 1 = *Almost all the time* to 5 = *Never*. These two items were reverse coded, and all three items were standardized and then averaged into a measure of financial worry (*α* = .83). Two-level multilevel models with participants nested within couples showed that 37% of the variance in financial worry was between couples and 63% was between participants. This suggests that two partners were not necessarily equally worried about their financial situation.

Participants completed the 16-item Couples Satisfaction Index (Funk & Rogge, 2007; e.g., “In general, how satisfied are you with your relationship?”) on 6-point scales. Items were averaged to capture overall relationship satisfaction (*α* = .96).

Then, they recalled a specific interaction: “Next, we would like to get an idea of one specific disagreement you experienced in your romantic relationship. Please think of a time in the past 1–2 weeks that you and your partner disagreed about something in your relationship.” Participants contacted in the initial recruitment wave also indicated keywords that would jog their partner’s memory and rated how likely they thought it would be that their partner would remember the incident, with 95.9% of the initial participants judging it to be likely or very likely that their partner would remember. Partners were shown the keywords and rated how likely it is that they remember the correct disagreement and 96.9% of the partners judging it likely or vey likely that they remembered the correct event. All participants also reported the topic of the disagreement by selecting one or more topics from a list: 10.8% selected “Amount of Time spent together”, 24.7% selected “Household chores”, 20.6% selected “Financial decisions and money habits”, 16% selected “Parenting decisions”, 9.3% selected “Demonstrations of affection”, 16% selected “In-law/Family Disagreements”, and 17.5% stated that the topic of their disagreement did not fall under one of the categories listed. The list of topics was adapted from the long version of the Couple Satisfaction Index (Funk & Rogge, 2007), with additional topics from a study listing common disagreements between couples (Papp et al., 2009).

Participants then rated perceived supportive behavior during the disagreement (“Now please tell us how you felt when having this disagreement…”) on four items (van Erp et al., 2011; “I felt my partner treated me with respect.”, “My partner understood my feelings.”, “I felt supported by my partner.”, “I felt I was valued by my partner.”) on scales from *Not at all* (1) to *The whole time* (5). Items were aggregated (*α* = .93). They rated only perceived behavior, not their own behavior.

### Plan of analysis

**Figure 1.**
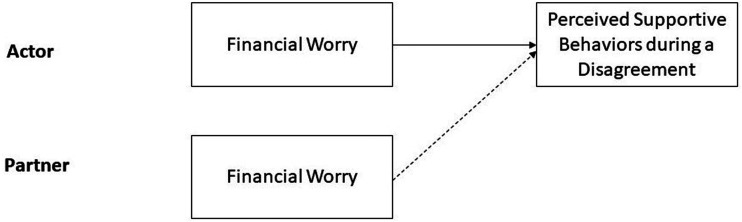
Conceptual model of the actor-partner model of financial worry (Study 1).

We first examined descriptive statistics and Pearson bivariate correlations with perceived supportive behaviors during the disagreement. We then examined the role of financial worry in perceived supportive behaviors within an Actor-Partner Interdependence framework (Kashy & Kenny, 1999). In other words, we examined both actor’s and partner’s financial worry as a predictor in the model. Dyads were treated as indistinguishable to allow for the inclusion of same-sex couples (i.e., every participant is both an Actor and a Partner). In a two-level multilevel model where participants were nested within dyads, perceived responsiveness of the partner during the disagreement was regressed on ratings of financial worry (Actor and Partner ratings). Figure 1 depicts the conceptual model.

In a follow-up regression model, we added demographic variables (age, gender, marital status, relationship length, income) as covariates, to account for the possibility that financial worry is simply a proxy for demographic differences. In another follow-up model, we examined both actor’s and partner’s relationship satisfaction as covariates, to account for the possibility that a link between financial worry and perceived partner behavior may have been due to relationship satisfaction as a common third variable.

## Results

Means and correlations are portrayed in Table 1. Across all participants, greater financial worry correlated with perceiving the partner as less supportive during the disagreement, and with being overall less satisfied in the relationship.

**Table 1.** Descriptive statistics and correlations (study 1).

	*M*	*SD*	Correlations (Pearson’s *r*)	
			Relationship Satisfaction	Perceived Supportive Behaviors
Age in years	37.91	10.13	−.18*	−.12
Gender	51% women		−.11	−.17*
Relationship Length in months	140.95	95.01	−.10	−.10
Legal marital status	51% married		.02	.04
Income	4.96	2.58	.04	.19*
Income Volatility	2.24	1.70	−.06	−.07
Financial Worry	0	.86	−.23*	−.25^**^
Relationship Satisfaction	4.97	.83	1	.38^**^
Perceived Supportive Behaviors	2.98	1.24	.38^**^	1

Note. *N* = 194. ** *p* < .01, * *p* < .05. Note that unmarried couples were mostly in common-law marriages. Income assessed in 11 categories. Income volatility assessed on a 7-point scale. Financial worry aggregated three standardized scales (10 pt, 7 pt and 5 pt scales). Relationship satisfaction assessed on a 6-point scale. Perceived supportive behaviors during the disagreement assessed on a 5-pt scale.

We next examined the role of the financial worry in perceived responsive behaviors in regressions following the Actor-Partner Interdependence framework (Kashy & Kenny, 1999). Actors perceived the partner’s behavior as less responsive if they themselves were more financially worried (Actor effect), *Unstandardized B* = −.37, *SE* = .10, _95%_*CI*[−.58; −.17], *t*(191) = −3.68, *p* < .001, but not if their partner was more financially worried (Partner effect), *Unstandardized B* = .04, *SE* = .10, _95%_*CI*[−.16; 0.24], *t*(191) = .41, *p* = .686. Adding demographic control variables (age, gender, marital status, relationship length, income) to this regression model as covariates did not change the results: *Unstandardized B* = −.30, *SE* = .12, _95%_*CI*[−.53; −.06], *t*(170.34) = −2.52, *p* = .013 (Actor effect), *Unstandardized B* = −.04, *SE* = .11, _95%_*CI*[−.26; .18], *t*(170.29) = −.35, *p* = .724 (Partner effect), see online supplements on OSF for all coefficients. Controlling for both actor’s and partner’s relationship satisfaction also did not change the results: Actors perceived the partner’s behavior as less supportive if they themselves were more financially worried (Actor effect), *B* = −.28, *SE* = .10, _95%_*CI*[−.48; −.09], *t*(189) = −2.82, *p* = .005, and if they were satisfied with their relationship overall, *B* = .42, *SE* = .10, _95%_*CI*[.22; 0.63], *t*(189) = 4.12, *p* < .001. Actors did not perceive the partner’s behavior as more or less supportive if their partner was more financially worried (Partner effect), *B* = .09, *SE* = .10, _95%_*CI*[−.11; 0.28], *t*(189) = .88, *p* = .378, or if their partner was more satisfied with the relationship, *B* = .09, *SE* = .10, _95%_*CI*[−.11; 0.30], *t*(189) = .89, *p* = .375. Thus, the link between financial worry and perception of partner’s behavior was not explained by demographic differences or worse overall relationship appraisal.

In sum, financial worry was associated with seeing one’s partner as behaving less respectful, understanding, and supportive during a recent disagreement. While this study cannot speak to whether these perceptions were biased or accurate, this finding supports the hypothesis that financial worry appears to be associated with perception of the partner’s positive relationship behaviors (H1).

## Study 2

In the next study we examined perception of both positive and negative behaviors (H1, H2a, H2b). In a recent set of studies, Joel and colleagues (2022) created an extensive list of concrete relationship behaviors that are overtly performed. Thus, these behaviors can be perceived by the partner, generated from multiple relationship samples’ reports of commonly performed behaviors in relationships. We assessed both self-reported and perceived behaviors using this list which comprises a variety of positive and negative behaviors. This allowed us to examine whether the link between financial worry and perceived behaviors may constitute a bias in perception. Applying the Truth and Bias Model (e.g., Stern & West, 2018; West & Kenny, 2011), we first examined the overall bias in perception of positive and negative relationship behaviors, and then examined whether financial worry moderates any potential perceptual bias. Data collection plan, methods, and analyses were preregistered: https://aspredicted.org/67Y_9V1. Unabbreviated materials, data and syntax are available on OSF: https://osf.io/89trb/. We report all manipulations, measures, and exclusions in this study. We also ran two pilot studies assessing one person’s report of financial worry and relationship behaviors. Results replicated those of the dyadic study reported below. Data and results summaries are available in online supplements (Pilot 1: https://osf.io/n3mzs/; Pilot 2: https://osf.io/zq2g7/).

### Method

#### Participants

A total of 252 participants in relationships were recruited from Prolific Academic and compensated with $5 (prorated for $15/hour). Participants passed Prolific data quality checks and a reCAPTCHA check before starting the study, as well as an attention check during the study. Only participants who stated they had a partner who also had an account on Prolific Academic were recruited for the online survey. They entered their partner’s contact information, who were then invited to participate as well. For our analyses, we designated the first partner to sign up for the study as “actor” and their corresponding partner as “partner”. We considered only data sets where both partners completed the survey, resulting in a final sample of 198 participants (99 couples) which the analyses below are based on. No participants were excluded from this dataset. Less than .01% of data were coded missing. Simulated power analyses (Lane & Hennes, 2018) suggest that this sample has 88.7% power to detect a small effect (*b* = .20), and over 99% power to detect a medium (*b* = .35) or larger effect in the context of a multilevel model with two predictors (e.g., a Truth and Bias Model).

The sample included 94 women and 104 men, ranging in age from 22 to 85 years (*M* = 43.14 years, *SD* = 11.81, *Mdn* = 39). The majority of the sample was White (87.9% White, 3.5% Black, 5.6% of Asian descent, 3% mixed or multiracial). Most participants were from the U.K. (81.7%), with the remaining participants from the US (16.7%) and Canada (1.6%). The majority (90.9%) considered themselves heterosexual or straight, 2.4% considered themselves gay, 1.2% lesbian, 5.2% bisexual, 0.5% bicurious. Of this sample, 96 (97%) of the couples were married, 2 couples were engaged and living together, 1 couple were dating and not living together. Average relationship length was 207.07 months, or 17 years (*SD* = 11.07 years). We did not assess student status or disability status. Participants’ annual gross income ranged from “under $10,000” to “$100,000 or more”, with the median income category being “$40,000–$50,000” and the income being normally distributed around this category as well. For comparison, the average annual income for U.S. workers is approximately $60,575 and the median income is $56,420 (DeMarco, 2023), suggesting that our sample earned just a little below the national average.

#### Procedure

Participants completed a brief demographic survey (age, gender, ethnicity, relationship status, and relationship length) and reported on their financial situation (income, income volatility), as in Study 1. Then participants were asked to think of the past week, defined as Sunday to Sunday; data was collected on a Sunday and the subsequent Monday. Defining the time frame ensured that both partners were thinking of the same time period.

Participants reported their financial worry during this time period on a 20-item Financial Worry Scale which assesses both negative emotions and cognitions (e.g., “I felt anxious when I thought about my finances”, “I cannot stop thinking about my finances”, de Bruijn & Antonides, 2020; α = .95) on a Likert scale ranging from 1 = *Completely Disagree* to 5 = *Completely Agree.* Two-level multilevel models with participants nested within couples showed that 50% of the variance in financial worry was within couples and 50% was between couples. This suggests that while two partners within the same couple did not necessarily worry equally about their financial situation, there was a strong overlap in extent of worry between partners of the same couple.

Next, we assessed reports of relationship behaviors in the same time period with a 33-item relationship behavior scale for participants’ own and their partner’s behavior (Joel et al., 2022) on a 4-pt scale (*1 = Not in the last week, 2 = Once in the last week, 3 = Several times in the last week, 4 = All the time in the last week*). The scale included 18 positive behaviors (*told my partner that I appreciate him/her, complimented my partner, been physically affectionate, been willing to try new things, expressed sexual interest, arranged fun things to do together, made an effort to clean up after myself, initiated sexual activities, done something nice for my partner, told my partner how much he/she means to me, talked about issues in our relationship, took care of things so my partner could relax, made an effort to look good for my partner, helped my partner solve a problem, initiated discussions to talk things over, helped without being asked, made an effort to spend time and do things with my partner, protected my partner from stress*) and 15 negative behaviors (*said something that hurt my partner’s feelings, demanded too much of my partner’s time or energy, expressed suspicion or distrust, avoided sexual activities, hid my feelings, been distracted or disengaged when my partner tried to talk to me, teased my partner in a mean, non-joking manner, refused to consider my partner’s point of view, been flirty with someone else, neglected chores, neglected my partner’s sexual needs, been too busy to spend quality time, been unwilling to discuss issues, done small things that irritate my partner, bored my partner with mundane stories*). Ratings were averaged for the positive behaviors (*α* = .91) and the negative behaviors (*α* = .83), respectively. The order in which participants rated their own behaviors and perceived partner behaviors was counterbalanced, but order did not significantly affect behavior ratings, all *t*s < 1.69, *p*s > .093, and was not considered further.

### Plan of analysis

**Figure 2.**
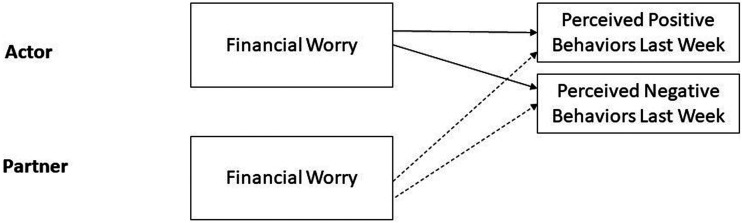
Conceptual model of the actor-partner model of financial worry (Study 2).

We first examined descriptive statistics and Pearson bivariate correlations with positive and negative relationship behaviors. We then examined the role of financial worry for perceptions within an Actor-Partner Interdependence framework (Kashy & Kenny, 1999). Dyads were treated as indistinguishable to allow for the inclusion of same-sex couples (i.e., every participant is both an Actor and a Partner). In a two-level multilevel model where participants were nested within dyads, perceived behaviors were regressed on each partner’s ratings of financial worry. In a follow-up regression model, we added demographic variables (age, gender, marital status, relationship length, income) as covariates. Figure 2 presents the conceptual model.

**Figure 3.**
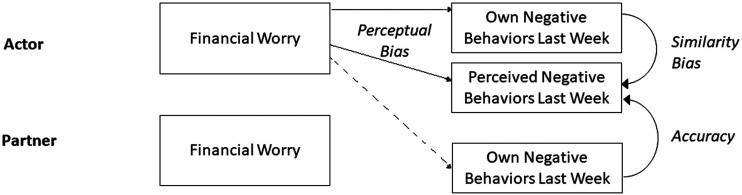
Conceptual model of the truth and bias model of financial worry (Study 2).

Then, in addition to examining direct links of financial preoccupation with perceived behaviors, we also examined the *bias* in perceived relationship behaviors using the Truth and Bias Model (West & Kenny, 2011; preregistered). First, in two-level multilevel models where participants were nested within dyads, we regressed actor’s perception of partner behavior on actor’s report of their own behavior and on partner’s report of their own behavior. In line with recommendations (West & Kenny, 2011), actor’s perception of partner behavior and actor’s report of their own behavior were centered by subtracting the grand mean of partner’s reports from each actor’s response, and partner’s reports of their own behaviors was centered by subtracting the scale mean from each partner’s response. This analysis tests the presence of directional bias while controlling similarity bias (link between actor’s own behavior and actor’s perceived behavior) and accuracy of reports (link between partner’s own behavior and actor’s perceived behavior). See Figure 3 for a conceptual model.

Finally, we examined whether greater financial worry was associated with greater bias in perceptions of the partner’s behavior (preregistered). Following Truth and Bias Model recommendations for testing moderation of bias, we regressed actor’s perception of their partner’s behavior on actor’s self-reported financial worry, actor’s report of their own behavior, partner’s report of their own behavior, and the two interaction terms with financial worry. In this model, the main effect of financial worry indicates moderation of the directional bias (West & Kenny, 2011).

### Results

Means and correlations are portrayed in Table 2. Across all participants, worry about finances was not linked with reporting performing more or less positive relationship behaviors oneself, *r* = −.06, *p* = .442 and was linked only marginally with perceiving fewer positive relationship behaviors from the partner, *r* = −.13, *p* = .064 (Table 2). However, correlations with negative relationship behaviors were significant: The more participants reported worrying about their finances, the more negative relationship behaviors they reported enacting themselves, *r* = .46, *p* < .001, and the more negative relationship behaviors they reported seeing their partner perform, *r* = .45, *p* < .001 (Table 2).

**Table 2.** Descriptive statistics and correlations (study 2).

	*M*	*SD*	Correlations (Pearson’s r)	
			Perceived Positive Behaviors	Perceived Negative Behaviors
Income	5.39	2.89	−.01	−.08
Income Volatility	2.19	1.63	.08	.13†
Financial Worry	2.50	.95	−.13†	.45^**^
Own Positive Behaviors	2.40	.59	.74^**^	−.10
Own Negative Behaviors	1.47	.40	−.21^*^	.68^**^
Perceived Positive Behaviors	2.24	.61	1	−.32**
Perceived Negative Behaviors	1.50	.43	−.32**	1

Note. *N* = 198. † *p* < .08, * *p* < .05, ** *p* < .01. Income assessed in 11 categories. Income volatility assessed on a 7-point scale. Financial worry assessed on a 5-pt scale. Relationship Behaviors assessed on a 4-pt scale.

We next examined the role of the financial worry for perceived behaviors in regressions following the Actor-Partner Interdependence framework (Kashy & Kenny, 1999). Actors perceived more negative behaviors if they themselves were more financially worried (Actor effect), *Unstandardized B* = −.18, *SE* = .03, *t*(177.63) = 5.63, *p* < .001, and perceived marginally more negative behaviors if their partner was more financially worried (Partner effect), *Unstandardized B* = .06, *SE* = .03, *t*(177.63) = 1.81, *p* = .072. Actors perceived positive behaviors similarly regardless whether they themselves were more financially worried (Actor effect), *Unstandardized B* = −.03, *SE* = .05, *t*(195) = −.61, *p* = .540, but perceived fewer positive behaviors if their partner was more financially worried (Partner effect), *Unstandardized B* = −.11, *SE* = .05, *t*(195) = −2.45, *p* = .015. Adding demographic control variables (age, gender, marital status, relationship length, income) to these regression models as covariates did not change the results for negative behaviors: *Unstandardized B* = .17, *SE* = .03, *t*(171.52) = 5.13, *p* < .001 (Actor effect), *Unstandardized B* = .04, *SE* = .03, *t*(169.72) = 1.28, *p* = .204 (Partner effect); see online supplements on OSF for all coefficients. Adding demographic control variables did not change the results for positive behaviors: *Unstandardized B* = −.07, *SE* = .05, *t*(175.98) = −1.46, *p* = .147 (Actor effect), *Unstandardized B* = −.14, *SE* = .05, *t*(175.59) = −2.81, *p* = .005 (Partner effect); see online supplements for all coefficients. In sum, actors perceived their partner as acting more negatively the more they themselves were worried about finances, and perceived their partner as acting less positively the more their partner was worried about finances.

We next examined potential bias in participants’ perception using the Truth and Bias paradigm (West & Kenny, 2011; preregistered), where actors’ and partners’ own behavior reports are controlled. Table 3 presents coefficients. The first model showed a significant intercept for positive relationship behaviors, suggesting a significant underestimation bias, with actors perceiving fewer positive behaviors from their partner than their partners reported performing. For negative relationship behaviors, the intercept was not significant, indicating no significant directional bias. In the second model, financial worry was marginally significantly associated with perceiving fewer positive behaviors and was significantly associated with perceiving more negative behaviors, suggesting that those who worried more about their finances in the last week overestimated the frequency of negative behaviors performed by their partner in the last week (Table 3).

**Table 3.** Multilevel Regression model coefficients for the Truth and Bias Models (Study 2).

	Perceived Positive Behaviors				Perceived Negative Behaviors			
	*B*	*SE*	*t*	*p*	*B*	*SE*	*t*	*p*
Model 1								
**Intercept** *(i.e., directional bias)*	**−.16**	**.03**	**−5.81**	**<.001**	**.03**	**.02**	**1.39**	**.167**
Actor’s own behaviors *(i.e., similarity bias)*	.26	.05	5.03	<.001	.17	.06	2.68	.009
Partner’s own behaviors *(i.e., accuracy)*	.66	.05	12.90	<.001	.66	.06	10.59	<.001
Model 2								
Intercept	−.16	.03	−6.01	<.001	.04	.02	1.45	.150
**Financial Worry**	**−.05**	**.03**	**−1.72**	**.089**	**.08**	**.03**	**2.92**	**.004**
Own behaviors	.63	.05	12.65	<.001	.58	.07	8.30	<.001
Partner’s own behaviors	.28	.05	5.60	<.001	.16	.06	2.52	.013
Own behaviors × Worry	−.19	.05	−3.86	<.001	−.002	.07	−.03	.973
Partner’s own behaviors × Worry	.13	.05	2.43	.017	−.04	.06	−.60	.550

Note. ** *p* < .01, * *p* < .05. Participants and partner’s self-reported behaviors were matched to the outcome variables (positive and negative behaviors, respectively).

Overall, this study suggests a bias towards seeing the relationship in a gloomier light for those who are preoccupied with negative thoughts about their finances, supporting Hypothesis 2b rather than Hypothesis 2a. Participants who reported having been more worried about their finances in the last week perceived more negative relationship behaviors from their partner after controlling for the negative behaviors their partner reported performing. On the other hand, financial worry appeared to be more weakly associated with positive relationship behaviors and to be more indirectly associated: An individual’s financial worry was associated with their partner seeing fewer positive behaviors, even if they themselves did not report performing fewer behaviors.

## Discussion

Financial hardship can affect relationships negatively (Dew, 2008; Falconier & Jackson, 2020; Jackson et al., 2023; LeBaron-Black et al., 2022), in line with theoretical models such as the Family Stress model (Casaburo et al., 2023; Conger et al., 1994, 2010) and the Vulnerability-Stress-Adaptation model (Karney & Bradbury, 1995). Both these models posit that external stressors (such as financial hardship and worry) can affect close relationship functioning. The present research examines one avenue by which financial worry might impact relationships: perceptions of partner behaviors. In the first study we examined a specific instance, namely recalled behaviors during a recent disagreement between partners. In the second study we examined a range of behavior recalled over a longer time frame. Worrying about finances was associated with perceiving one’s partner as less supportive (Study 1) and was associated with perceiving more negative behaviors (teasing, neglect, or expressing distrust) from one’s partner over the course of a week (Study 2). Thus, findings conceptually replicate across two instances that examine perceived behavior over different time frames. The second study further expanded upon the first study by examining reports of participants’ own and their perception of their partners’ behavior from both individuals in the relationship, suggesting that the increase in negative behaviors perceived by the financially worried participant was not corroborated by their partner.

### Practical implications

An awareness that financial worries tend to co-occur with a negatively biased perception of relationship behaviors might benefit couples by encouraging a critical view of their perception of their own and their partner’s behaviors. Knowing that financial stress and worse mood might colour perception might encourage people to examine whether perceived negative behaviors are indeed negative or whether they might be interpreted in a more beneficial light – or might encourage them to explicitly look for supportive behaviors they might have missed. When understanding the possibility of bias and that behaviors being interpreted more negatively than they were intended, people might also attempt to reduce ambiguity in their own behaviors towards their partner, leaving them less open to negative interpretations. More generally, these studies, along with many others (Karney & Bradbury, 1995; Conger et al., 2010; Dew, 2008), suggest that reducing external stressors such as financial worries might benefit relationships.

### Theoretical contributions

The present results are consistent with theories explaining how external stressors might affect relationships and families (Conger et al., 1994, 2010; Karney & Bradbury, 1995). Specifically, the Family Stress model (Conger et al., 1994, 2010) posits that economic pressure creates conflict between partners due to both partner’s psychological distress. In line with this theory, our studies showed patterns of behavior and perception bias that suggest worse mood and greater distress (perceiving more negative and fewer positive behavior) rather than patterns that would suggest cognitive distraction (perceiving fewer behaviors overall). Though we did not assess frequency of conflict and rather focused on specific behaviors, we argue that presence of more negative relationship behaviors is itself a form of conflict between partners (e.g., see Rogge & Bradbury, 1999; Murray et al., 2003). The present studies are also in line with the Vulnerability-Stress-Adaptation model (Karney & Bradbury, 1995) which posits that stressful events affect marital quality via (mal)adaptive processes. The negative bias in perception of the partner’s behavior is such a maladaptive process linked with external stressors (i.e., financial worry), and the link with relationship satisfaction and perceived supportive behaviors (Study 1) further fits the Vulnerability-Stress-Adaptation model’s proposed link between (mal)adaptive processes and relationship quality.

Beyond contributions to theory, the present studies extend the growing literature showing that financial stressors and financial hardship can be detrimental to relationship quality (Dew, 2008; Falconier & Jackson, 2020; Jackson et al., 2023; Kelley et al., 2018; LeBaron-Black et al., 2022; Totenhagen et al., 2019) by examining their links with perceptions of positive and negative behaviors specifically. The present studies also replicate and extend recent work showing that life event stressors are associated with a perception bias for *negative *relationship behavior (Neff & Buck, 2023). The present studies replicate this finding in the financial domain, showing that feeling *subjectively* stressed and worried about finances is also associated with biased perceptions of *negative *relationship behaviors.

### Limitations and future directions

A major limitation of the present studies is their correlational design. We assessed financial worry at the same time as reports of relationship behaviors. Thus, the direction of the association cannot be ascertained (see Saxey et al., 2023, for a discussion of bi-directionality between finances and relationship outcomes). It is also possible that there are variables such as mental health concerns that affect both financial worry and perception of relationship behaviors. Future research might assess financial and relational variables in separate surveys or assess relationship behaviors over multiple assessments (similar to Neff & Buck, 2023) to draw directional conclusions. Future research might also examine the role of financial worry experimentally. Financial stress and associated cognitive loads can be shifted: For example, a supplemental income intervention increased memory performance among elderly participants (Aguila & Casanova, 2020), and low-income workers performed better on Stroop tasks when they were tested after their payday, rather than before their payday (Mani et al., 2020). Future studies might take a more experimental approach by manipulating people’s actual or subjective financial situation to examine subsequent shifts in perception of relationship behaviors.

Another limitation is the measure of relationship behaviors. We assessed ratings of supportive behaviors during one interaction (van Erp et al., 2011; Study 1) and of the frequency of a range of positive and negative behaviors over the course of one week (Joel et al., 2022, Study 2). Both types of assessments were only snapshots of behaviors participants experienced in their relationships rather than an exhaustive assessment of all the behaviors participants experienced. Furthermore, in cases where a list of possible behaviors is provided to participants (Study 2), behaviors of a certain kind might seem more familiar than others, driving any associations. Dual-process accounts of recognition memory (e.g., Mandler, 1980) hold that such familiarity, which reflects the strength of activation for a particular item, may be responsible for false positive responses (Yonelinas, 2002). To prevent familiarity from affecting participants reports and to assess a wider range of possible behaviors, future research could ask participants to list behaviors in an open-ended fashion rather than self-report behaviors via a prepared list. However, such alternative measures have different problems such as underreporting due to the relative difficulty of retrieval recall compared to recognition-recall (Begg et al., 1989).

The current studies also highlight the difficulty of determining which behaviors were ‘truly’ enacted. When participants report performing more positive behaviors than their partners report seeing them perform, who is correct? Although behavior descriptions were designed to be concrete and observable (Joel et al., 2022), there is flexibility in how behaviors are interpreted. For example, a person observing that their partner has gotten a haircut might be interpreted as a compliment, criticism, or as neutral statement. The perception bias found in the current studies might suggest that having a lot of financial worries on one’s mind might change the interpretation of ambiguous statements – or might affect the notice and recall of behaviors. Future research could tease possible mechanisms apart by reducing the ambiguity of relationship behaviors and assessing behaviors objectively by observing relationship behaviors in the lab, introducing impartial third observers, or assessing behaviors via standardized hypothetical scenarios.

### Constraints on generality

The sample was primarily White, married or common-law, and not limited to people who are struggling financially. Participants reported a range of different financial situations and income, which was the reason we focused on the *subjective* experience of financial worry, which can be present at any income level (de Bruijn & Antonides, 2020; Johar et al., 2015; Magwegwe et al., 2023). It is notable that actual income or even income regularity was not associated with perceived behaviors in either study. It is possible that the link between subjective financial worry and relationship behaviors is stronger among people living in financial insecurity. It is also possible that the link between subjective financial worry and relationship behaviors is weaker in countries with more generous social safety nets or in cultures that share finances among a greater number of family members other than the nuclear family or couple.

The samples gathered in both studies were overwhelmingly White. This sample make-up limits the generalizability of our results, as those of different races differ significantly in matters of financial stress and relationships. For example, White participants may experience less financial stress (Lee et al., 2022), and report more symptoms of cognitive and emotional impact of financial stress (Marshall et al., 2022).

Participants in both studies were in long-term, committed relationships. Participants in the first study were either married or in common-law relationships and participants in the second study were married. Sharing finances as part of a marriage or common-law marriage might lower financial stress, as a result of pooling income and divided costs (see Dew, 2008, for a discussion). The overall financial stress level and the link between financial worries and perception of relationship behaviors might be different among couples that have been dating short-term or who do not live together.

### Conclusions

Financial stress impairs optimal functioning. Two dyadic studies showed that people’s worry about finances was associated with perceiving less supportive behaviors from their partner in a specific situation and was associated with perceiving more negative behaviors, such as showing lack of care via teasing, neglect, or expressing distrust, over the past week. These findings underline the role of financial concerns in close relationships and shows how recurring, intrusive, and negative thoughts about money might make people see the relationship – and their own and their partner’s behavior – through dark-tinted glasses.
